# Influence of Social Isolation During Prolonged Simulated Weightlessness by Hindlimb Unloading

**DOI:** 10.3389/fphys.2019.01147

**Published:** 2019-09-13

**Authors:** Candice G. T. Tahimic, Amber M. Paul, Ann-Sofie Schreurs, Samantha M. Torres, Linda Rubinstein, Sonette Steczina, Moniece Lowe, Sharmila Bhattacharya, Joshua S. Alwood, April E. Ronca, Ruth K. Globus

**Affiliations:** ^1^Space Biosciences Division, NASA Ames Research Center, Moffett Field, CA, United States; ^2^KBR, Houston, TX, United States; ^3^Universities Space Research Association, Columbia, MD, United States; ^4^Blue Marble Space Institute of Science, Seattle, WA, United States; ^5^Department of Obstetrics and Gynecology, Wake Forest School of Medicine, Winston-Salem, NC, United States

**Keywords:** hindlimb unloading, microgravity model, bone loss, muscle atrophy, immune system, spaceflight, social isolation, HPA axis

## Abstract

The hindlimb unloading (HU) model has been used extensively to simulate the cephalad fluid shift and musculoskeletal disuse observed in spaceflight with its application expanding to study immune, cardiovascular and central nervous system responses, among others. Most HU studies are performed with singly housed animals, although social isolation also can substantially impact behavior and physiology, and therefore may confound HU experimental results. Other HU variants that allow for paired housing have been developed although no systematic assessment has been made to understand the effects of social isolation on HU outcomes. Hence, we aimed to determine the contribution of social isolation to tissue responses to HU. To accomplish this, we developed a refinement to the traditional NASA Ames single housing HU system to accommodate social housing in pairs, retaining desirable features of the original design. We conducted a 30-day HU experiment with adult, female mice that were either singly or socially housed. HU animals in both single and social housing displayed expected musculoskeletal deficits versus housing matched, normally loaded (NL) controls. However, select immune and hypothalamic-pituitary-adrenal (HPA) axis responses were differentially impacted by the HU social environment relative to matched NL controls. HU led to a reduction in % CD4^+^ T cells in singly housed, but not in socially housed mice. Unexpectedly, HU increased adrenal gland mass in socially housed but not singly housed mice, while social isolation increased adrenal gland mass in NL controls. HU also led to elevated plasma corticosterone levels at day 30 in both singly and socially housed mice. Thus, musculoskeletal responses to simulated weightlessness are similar regardless of social environment with a few differences in adrenal and immune responses. Our findings show that combined stressors can mask, not only exacerbate, select responses to HU. These findings further expand the utility of the HU model for studying possible combined effects of spaceflight stressors.

## Introduction

The spaceflight environment poses significant challenges to multiple organ systems. Microgravity exposure leads to a cephalad fluid shift (Foux et al., 1976; Thornton et al., 1987) as well as osteopenia (Lang et al., 2004) and sarcopenia (LeBlanc et al., 2000). Spaceflight alters electrolyte balance (Vorobiev et al., 1995) as well as endocrine (Stowe et al., 2003, 2011), vision (Zhang and Hargens, 2018) and baroreflex processes (Fritsch et al., 1992; Buckey et al., 1996; Meck et al., 2001). Cardiovascular changes include stiffening of the carotid arteries as seen in astronauts returning from a 6-month ISS mission (Hughson et al., 2016) and transient changes in heart rate variability (Yamamoto et al., 2015). Spaceflight also may lead to CNS changes including a decline in cognitive performance (Eddy et al., 1998). Immune system dysregulation also occurs, characterized by altered immune cell function and reactivation of viruses (Crucian et al., 2015, 2018; Mehta et al., 2017). These findings from astronauts are the main motivation to study rodents flown into space with the rationale that rodents are used extensively to model human conditions on Earth and may prove useful for understanding mechanisms and anticipating human responses to spaceflight. However, limited opportunities for rodent spaceflight studies, along with other challenges of conducting microgravity experiments, stimulated interest in using ground-based rodent models of weightlessness.

The rodent hindlimb unloading (HU) model was developed initially to simulate the responses of the musculoskeletal system to microgravity (Wronski and Morey-Holton, 1987; Morey-Holton et al., 2005; Globus and Morey-Holton, 2016). To date, its use has expanded to study the responses of other organ systems to spaceflight, including immune, cardiovascular and central nervous system (Globus and Morey-Holton, 2016). The HU model also is utilized to study disorders on Earth such as osteopenia and sarcopenia because features of the HU model mimic bedrest and disuse. Initial efforts to generate a rodent model for musculoskeletal unloading entailed use of a back harness for inducing a cephalad fluid shift and musculoskeletal disuse of the hindlimbs in singly housed animals (Morey, 1979), which was then developed further to introduce tail suspension which improved outcomes (Wronski and Morey-Holton, 1987). This system employs non-invasive attachment of the tail via orthopedic traction tape onto a pulley system that allows for elevation of the hindlimbs; application with the custom-designed cage enables free movement of the animal about the cage (Morey-Holton and Globus, 2002; Morey-Holton et al., 2005). Since then, other variants of the HU system emerged and include partial unloading of all four limbs using a custom body harness (Wagner et al., 2010; Ellman et al., 2013; Mortreux et al., 2018), various methods to induce unloading such as surgical piercing of the tail, alternative taping methods, simplified cage systems that restrict movement, and group housing (Ferreira et al., 2011; Maurel et al., 2016).

Single versus social housing of rodents under standard, ambulatory conditions, can differentially impact multiple organ systems (e.g., cardiovascular, immune, and central nervous system) known also to be affected by spaceflight (Beisel and Talbot, 1987; Schmitt and Schaffar, 1993; D’Aunno et al., 2003; Migeotte et al., 2003; Pierson et al., 2005; Hughson et al., 2016; Mehta et al., 2017). Social isolation causes anxiety and depressive-like behavior (Djordjevic et al., 2012), disrupted circadian rhythms (Spani et al., 2003), deficits in memory (McLean et al., 2010), activation of pro-inflammatory transcription factors and impairments in antioxidant responses in the brain (Djordjevic et al., 2015). Social isolation also can stimulate the hypothalamic-pituitary-adrenal (HPA) axis (Jessop and Bayer, 1989; Ros-Simo and Valverde, 2012; Shetty and Sadananda, 2017), the main neuroendocrine regulator of mammalian stress responses (Bellavance and Rivest, 2014). However, other studies report that social isolation can lead to decrements in circulating corticosterone levels (Djordjevic et al., 2012; Ieraci et al., 2016) although it is not clear whether these reflect an initial downregulation of HPA axis activation or simply the induction of a negative feedback loop after a state of activation. In addition, social isolation in rodents leads to impairments in immune function (Jessop and Bayer, 1989; Clausing et al., 1994; Shanks et al., 1994; Wu et al., 2000; Krugel et al., 2014).

Social isolation can be a confounding factor for interpreting experimental outcomes from both rodent spaceflight and ground-based studies. In rats, 9 days of spaceflight reduced periosteal bone formation and bone mass (femur dry weights) in singly housed animals but had minimal effect on bone formation or mass in group housed animals (Morey-Holton et al., 2000). In an interesting study, Tsvirkun et al. (2012) report that HU (in standard, single housing) or social isolation (without unloading) each can perturb blood pressure and heart rate levels.

The possible contribution of social isolation to the biological outcomes of HU has not been studied, leaving a number of knowledge gaps in our understanding of the model for simulating weightlessness. It is unclear whether social interactions (social versus single housing) during HU differentially impacts organ systems known to be responsive to HU. Rodent experiments on the International Space Station (ISS), particularly those making use of the NASA Rodent Research habitat, are conducted in group housed conditions whereas most HU studies conducted to date entail use of the standard, single housed HU model. Other groups have developed variants of the HU model that accommodate social housing in pairs (Ferreira et al., 2011; Lloyd et al., 2012; Maurel et al., 2016). However, characterization of these models in side-by-side studies with the traditional singly housed HU model have not been conducted. Hence, there is value to further developing and characterizing the socially housed HU model for simulating weightlessness to better match rodent habitat conditions on the ISS. Addressing these gaps in knowledge will lead to an improved understanding of the contribution of isolation to observed responses to HU, which in turn will improve the ability to extrapolate HU results to humans in space.

To address these knowledge gaps, we aimed to (1) develop a refinement to the NASA Ames HU system, which traditionally entails housing mice in isolation (Morey-Holton and Globus, 2002; Morey-Holton et al., 2005) to enable housing in social pairs and (2) determine the contribution of social isolation to the HU responses of various tissues. We hypothesized that relative to social housing, single housing exacerbates HU-induced dysfunction in select organ systems. To meet these aims and test the hypothesis, we performed a side-by-side comparison of this alternative social HU system versus the standard NASA Ames single housed design and evaluated the responses of select spaceflight-relevant tissues to HU.

## Materials and Methods

### Animals and Experiment Design

**FIGURE 1:**
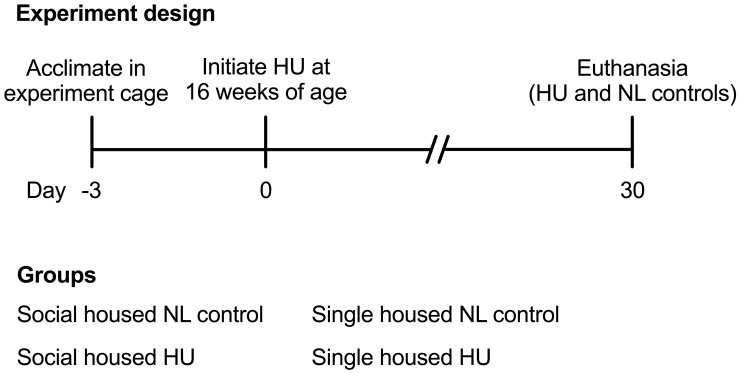
Experiment design and groups.

Animal experiments were carried out in accordance with the recommendations of Guide for the Care and Use of Laboratory Animals, 8th edition (National Research Council (U.S.) Committee for the Update of the Guide for the Care and Use of Laboratory Animals, Institute for Laboratory Animal Research (U.S.) and National Academies Press (U.S.), 2011). All animal experiments were conducted with prior approval from the NASA Ames Institutional Animal Care and Use Committee (IACUC). Sixteen-week old female mice that were to undergo HU were acclimated into HU cages (paired or single housing groups) 3 days prior to the onset of HU (Figure 1). Normally loaded (NL) controls were housed in standard vivarium cages either individually or in pairs. NL controls (either paired or single housing groups) were handled the same way as the HU groups except for the actual attachment of traction tape and tail suspension. Animals were bred at Ames and were wild type littermates of a cross between male mCAT mice [B6.Cg-Tg(CAG-OTC/CAT)4033Prab/J, Jackson Laboratories] (Schriner et al., 2005) and female C57BL/6NJ (Jackson Laboratories). After the acclimation period, animals were suspended onto the pulley apparatus via orthopedic traction tape as described previously (Wronski and Morey, 1982; Morey-Holton and Globus, 1998, 2002; Kondo et al., 2010). Responses to the social housing HU model also were evaluated in female C57BL/6J (Jackson Laboratories), a closely related yet distinct strain. Refer to Supplementary Section for further details on the C57BL/6J study. C57BL/6J and C57BL/6NJ mice differ in competency for nicotinamide nucleotide transhydrogenase (Nnt) expression (C57BL/6J are deletion mutants) (Freeman et al., 2006). Compared to animals with an intact Nnt gene, C57BL/6J mice have higher rates of H_2_O_2_ release, spontaneous oxidation of NADPH, and reduced ability to metabolize peroxide (Ronchi et al., 2013). C57BL/6J and C57BL/6NJ strains also display differences in metabolic phenotypes of muscle and other organs (Fisher-Wellman et al., 2016).

All animals were supplied with standard rodent laboratory chow (Purina, Cat# 5001) and water *ad libitum*. We attempted to measure food intake to gain insight into possible differences in body weight responses. However, accurate measurements could not be made since HU animals generated a substantial amount of food pellet fragments (not consumed) that inadvertently fell through the floor grate and could not be recovered. Enrichment consisted of cotton nestlets (Ancare, Cat# NES3600) refreshed daily. The nestlets were split (cut into two equal parts and stripped half of its thickness) to prevent the mice from building ramp-like structures to load the hindlimbs. Cage and bedding changes were performed weekly. Room temperature was maintained within 23–24°C (higher temperatures can be used) with a 12 h light-12 h dark cycle. After 30 days of HU, animals were euthanized via carbon dioxide inhalation followed by cervical dislocation. Blood was collected from vena cava. Soleus, spleen, and adrenal glands were collected and immediately weighed. Tibiae were recovered and then fixed in 4% paraformaldehyde in phosphate buffered saline solution (FD Neurotechnologies) for ∼24 h at 4°C then transferred to 70% ethanol (Sigma) for storage at 4°C until analysis. Unless otherwise stated, sample sizes (C57BL/6NJ) are as follows: Single NL = 8; Single HU = 6; Social NL = 12; and Social HU = 12. To mitigate any technical artifacts from variations in euthanasia times across groups, one animal from each group was euthanized sequentially, and the sequence repeated until all animals have been euthanized for any given day. All dissections were completed within a 7-h period.

### Social HU Cage Design and Construction

The social housing HU system for mice was generated using commercially available rat/guinea pig polycarbonate cages (Ancare, Cat# R20HT) with filter-top lids (Ancare, Cat# R20MBT). Holes were drilled on the walls of each cage to accommodate two food trays as well as the spouts of two water bottles. Food trays consisted of cryovial holders that were cut to fit into the short side wall of the cage (Nalgene, available from Thermo Fisher Scientific Cat# 5015-0002). The food tray was attached to each of the two short walls, 5.5 cm above the cage floor. A stainless steel mesh grate (Ancare, Cat# R20SSRWF) was laid out on the cage floor. Bedding was then placed such that it filled two thirds of the space between the cage bottom and the steel mesh grate. In our experience, adding more bedding is not ideal because animals tend to move the bedding to form mounds that come in contact with their hindlimbs. The grid of the ventilated cage lid was sawed out to create an open top. Using a drill, openings were created on one long side of the cage wall to install two spring attachments which were used to secure two water bottles (one for each animal). The spouts of these water bottles were routed into the cage 5.5 cm from the cage floor by drilling openings on the cage wall. Two rods were attached to the cage lids using binder clips to support two pulley systems (one for each animal).

The distance that the pulley apparatus can be moved by the animals along the suspended rod was adjusted carefully, using rubber washers to ensure that loading of the hindlimbs do not occur from contact with cage walls or the water sipper (Supplementary Figure S1). In addition, the two suspension rods were placed at an optimal distance from each other so that cephalad regions of the animals can come in contact while avoiding entanglement of the two sets of suspension apparatus (Supplementary Figure S1). To prevent loading of the hindlimbs while being weighed, HU animals were kept suspended on a small hook apparatus set over a lab animal balance as described in Morey-Holton and Globus (2002).

### Microcomputed Tomography

Microarchitecture of the cortical and cancellous compartments of the tibia was quantified by μCT using the SkyScan 1272 system (Bruker). For cancellous bone morphology, the proximal tibia was scanned at 3.5 μm resolution. A 400 μm region of interest was 3D reconstructed with a 450 μm offset from the proximal growth plate. This region was auto-contoured using SkyScan CT Analyzer (CTAn) Software (Bruker) to select for the cancellous region. Cancellous parameters such as Bone Volume (BV, mm^3^), Tissue Volume (TV, mm^3^), percent Bone Volume (BV/TV, %), Trabecular Spacing (Tb.Sp, mm), Trabecular Thickness (Tb.Th, mm), and Trabecular Number (Tb.N, 1/mm) were quantified following standard μCT guidelines (Bouxsein et al., 2010). For the analysis of cortical bone microstructure, the tibia was scanned at 12 μm resolution. A 300 μm region that was 200 μm proximal to the tibio-fibular junction (TFJ) was auto-contoured to analyze cortical bone parameters such as Marrow Area (Ma.Ar, mm^2^), Cortical Area (Ct.Ar, mm^2^), Endocortical Perimeter (Ec.Pm, mm), Periosteal Perimeter (Ps.Pm, mm), and Cortical Thickness (Ct.Th, mm).

### Flow Cytometry Analysis of White Blood Cells

Flow cytometric analysis of white blood cells (WBC) isolated from C57BL/6NJ was performed, as described previously (Paul et al., 2017). Briefly, peripheral blood was collected and centrifuged at 2000×*g* for 15 min and plasma removed. Red blood cell lysis buffer at 1X dilution (eBioscience) was added and cells were incubated for 10 min at room temperature on an orbital shaker followed by addition of 1X Phosphate Buffered Saline Solution (PBS, Thermo Fisher Scientific). Cells were then centrifuged at 500×*g* for 5 min at 4°C to pellet out the WBC, fixed in 2% paraformaldehyde (Thermo Fisher Scientific) for 15 min on ice, washed, and incubated with Fc block (CD16/32 Block) for 20 min, followed by probing with anti-CD45-FITC, anti-CD4-PE, anti-CD8a-PerCP, anti-CD11b-PECy5, and anti-Ly6G-PE antibodies (all purchased from Thermo Fisher Scientific) for 1 h at room temperature in the dark. Samples were then washed twice in 1X PBS, and acquired using a Guava Flow Cytometer (Millipore). Unstained and single stained compensation controls were used during acquisition and FlowJo software (version 10.3.0) utilized for cytometric analysis.

### Measurement of Plasma Corticosterone Levels

Peripheral blood was collected from the vena cava and introduced into K3 EDTA tubes (Sarstedt, Cat# 41.1395.105). Centrifugation was performed at 2000×*g* at room temperature for 10 min and plasma collected. Plasma was diluted 1:100 and analyzed using a corticosterone ELISA kit (Abcam, Cat# ab108821) according to manufacturer’s instructions.

### Statistical Analysis

Equivalence of variance was first evaluated by Levene’s test. If the variances were equal, two-way analysis of variance was performed. A Tukey *post hoc* test was employed when an interaction effect of *p* < 0.05 was observed. For multi-timepoint measures, repeated measures ANOVA was performed followed by Tukey *post hoc* test. If the variances were unequal, a two-factor linear model with interaction was employed treating the variance with a logarithmic transformation (Harvey, 1976; Cook and Weisberg, 1983; Aitkin, 1987), with a threshold of *p* < 0.05. Statistical analyses were performed using JMP software version 13.1.0 (SAS Institute Inc.). Data shown are mean ± standard deviation.

## Results

**FIGURE 2:**
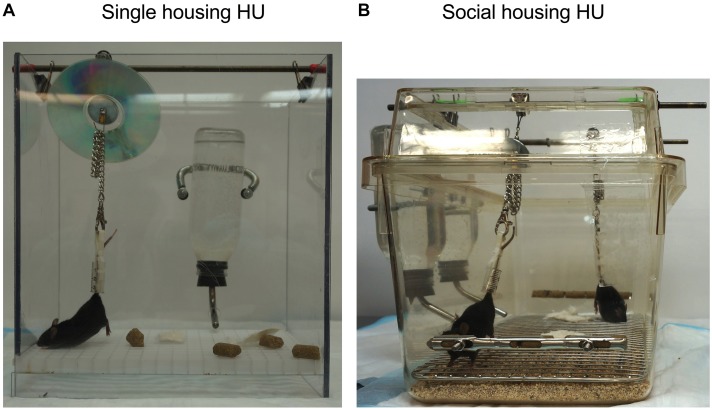
Side view of **(A)** single housing HU and **(B)** social housing HU cage design.

To test the hypothesis that social isolation impacts tissue responses to HU, we developed a refinement of the traditional NASA Ames HU cage design that allows paired housing of animals (Figure 2). We then performed a 30-day HU study under these social housing conditions, side-by-side with animals maintained according to the standard NASA single housing HU system (Figure 1). Animals selected for this study were female C57BL/6NJ mice shown previously to be sensitive to bone loss caused by HU (Sankaran et al., 2017). Controls (normally loaded, NL) were age- and sex-matched animals freely ambulating in standard mouse cages.

To begin to gain insight on whether responses to social housing HU were strain-specific, we also performed a separate 30-day HU experiment in socially housed female C57BL/6J mice (Supplementary Figures S3–S8), another commonly used strain to study musculoskeletal responses to disuse. Due to logistical constraints, this study had a smaller subset of experimental outcomes and only responses of socially housed NL controls and HU groups were evaluated.

**FIGURE 3:**
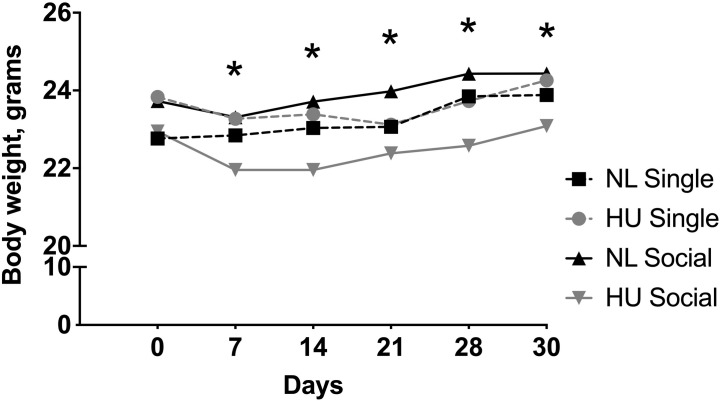
Body weights from day 0 to 30 of hindlimb unloading (HU) and corresponding normally loaded (NL) controls in singly and socially housed female C57BL/6NJ mice. ^∗^NL social and HU social groups display statistically significant differences at indicated days at *p* < 0.05 using two-way repeated measures ANOVA followed by Tukey *post hoc* test. Values are expressed as means. For clarity, error bars are omitted. Refer to Supplementary Table S1 for a full list of mean and SD values. Sample sizes: Single NL = 4; Single HU = 6; Social NL = 12; and Social HU = 12.

Body weights were measured at multiple time points during the HU period. In our experience, a modest weight loss (typically <10%) may occur within a week of HU. In C57BL/6NJ mice, there was a modest yet significant decline in body weights at days 7, 14, 21, 28, and 30 in the socially housed HU group compared to its NL control. At any given day of weighing, the magnitude of the difference in mean body weights of the socially housed HU group relative to its NL control was between 3 and 8%. There were no significant differences in body weights in the singly housed HU cohort versus singly housed NL controls at any time point (Figure 3 and Supplementary Table S1). We also looked into possible behaviors unique to the NL and HU social housing groups that could contribute to body weight differences. This was done by scoring select time periods in videos taken during the dark and light cycles (Supplementary Methods). Huddling, an adaptation that helps conserve body heat and therefore fat reserves, manifested in socially housed NL controls but not in socially housed HU animals. This was because physical contact between socially housed HU animals can only occur via cephalad regions. In addition, we observed a novel behavior wherein socially housed HU mice pass nestlets (enrichment) to or from their cagemates. Socially housed NL controls did not engage in this behavior (Supplementary Figure S2). In C57BL/6J mice, HU animals and their corresponding NL controls did not differ in body weights at any of the time points (Supplementary Figure S3 and Supplementary Table S2).

Muscle and bone, conventionally considered mechanosensitive tissues, are profoundly sensitive to unloading. Hence the impact of social isolation on the response of these tissues to HU was examined. The weight of the soleus, a postural muscle known to undergo atrophy in response to spaceflight and unloading (Musacchia et al., 1983; Steffen and Musacchia, 1986; Haida et al., 1989; Desplanches et al., 1990) was measured. Experimental groups had comparable body weights at the onset of the 3-day acclimation period in assigned cages. However, there was a trend (non-significant) for body weight differences (0.8–1.0 g) across groups at the onset of HU. To account for these slight differences, tissue weights were normalized to body weights by dividing tissue weight by the body weight at euthanasia. Use of actual tissue weights did not change the main results or interpretation (Refer to Supplementary Tables S3, S4 for mean and SD of actual tissue weights).

**FIGURE 4:**
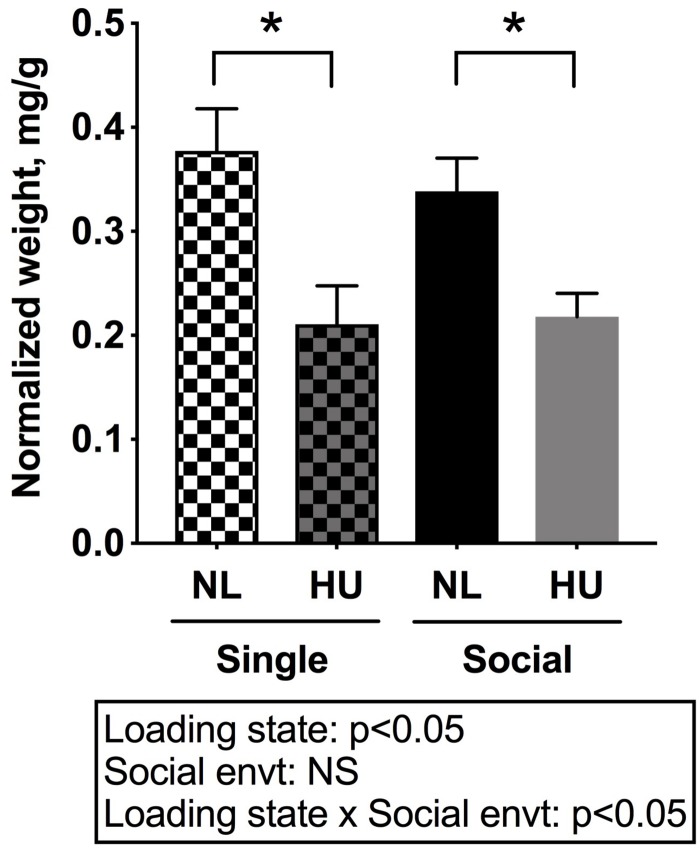
Soleus weights normalized to day 30 body weights of C57BL/6NJ mice. Social envt: Social environment. ^∗^Statistically significant at *p* < 0.05 using two-way ANOVA followed by Tukey *post hoc* test. NS: Not significant.

In C57BL/6NJ mice, HU led to decrements in normalized soleus weight (Figure 4), whether the mice were housed singly or socially. The magnitudes of muscle atrophy between single and social housing HU cohorts were 44 and 36% respectively. There were no differences in normalized soleus weights between the single and social HU groups. In addition, values for singly and socially housed NL controls were comparable (Figure 4). In C57BL/6J mice, social housing HU also led to the expected decrement in normalized soleus weight (Supplementary Figure S4).

**FIGURE 5:**
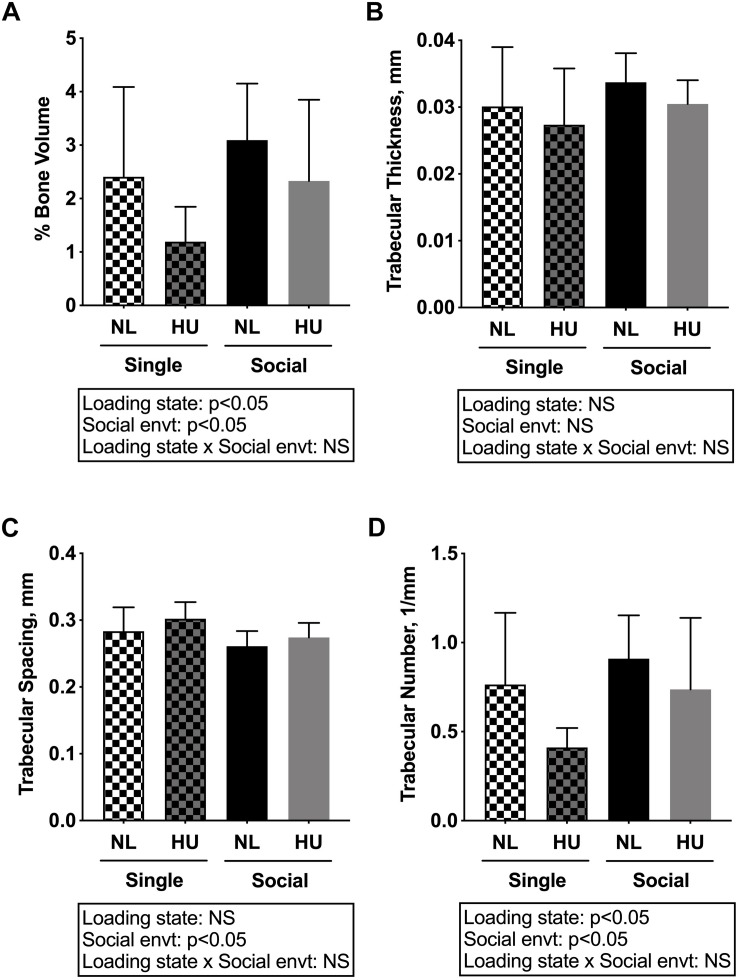
Microcomputed tomography (μCT) analysis of cancellous compartment of proximal tibiae from C57BL/6NJ mice. **(A)** % Bone Volume, % BV/TV; **(B)** Trabecular Thickness, Tb.Th; **(C)** Trabecular Spacing, Tb.Sp; and **(D)** Trabecular Number, Tb.N. Two-way ANOVA was performed. NS, not significant.

**FIGURE 6:**
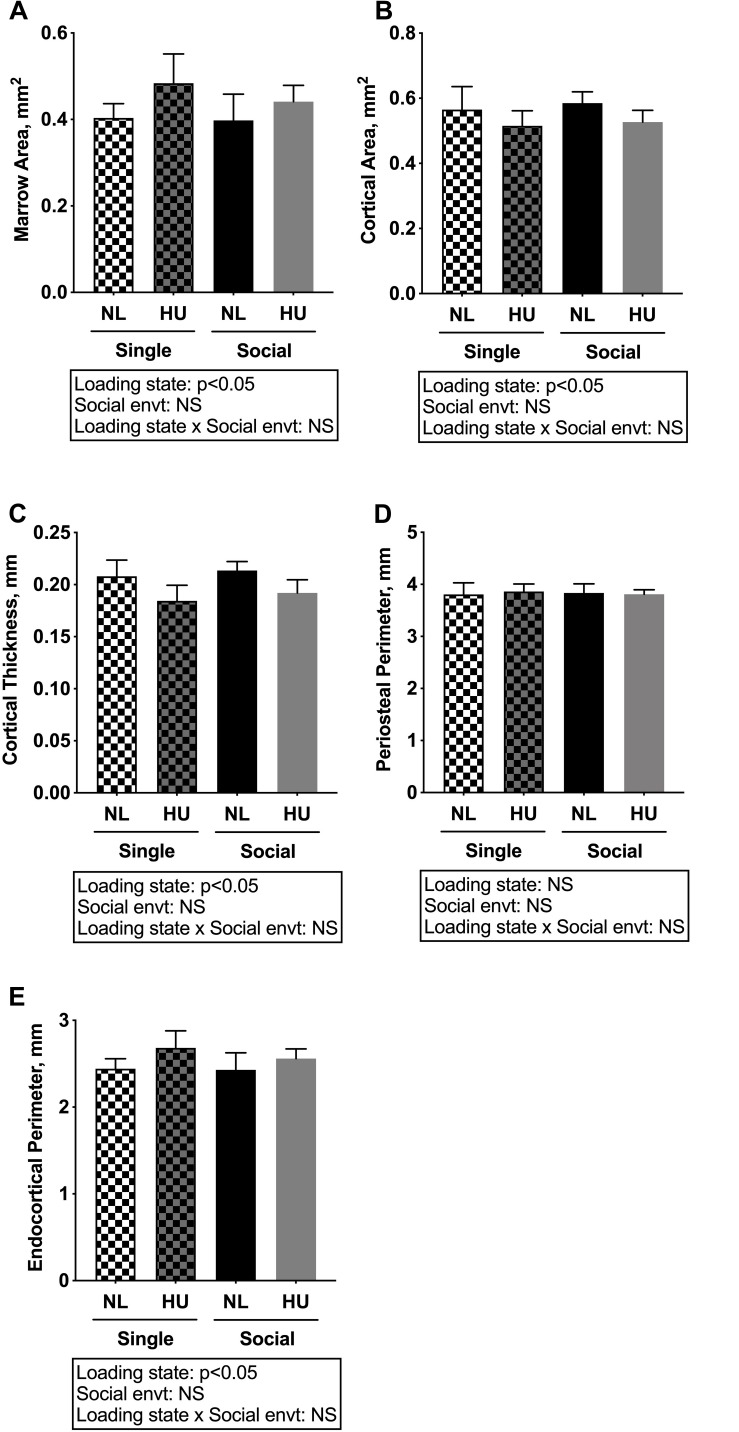
Microcomputed tomography analysis of cortical compartment of tibiae from C57BL/6NJ mice. **(A)** Marrow Area, Ma.Ar; **(B)** Cortical Area, Ct.Ar; **(C)** Cortical Thickness, Ct.Th; **(D)** Periosteal Perimeter, Ps.Pm; and **(E)** Endocortical Perimeter, Ec.Pm. Two-way ANOVA was performed. NS, not significant.

Microcomputed tomography (μCT) analyses were performed to determine the contribution of social isolation to HU-induced deficits in structure of cancellous (Figures 5A–D) and cortical (Figures 6A–E) bone compartments. As expected, in C57BL/6NJ mice HU led to deficits in some cancellous structural parameters, specifically % BV/TV (Figure 5A) and Tb.N (Figure 5D). Tb.Th (Figure 5B) and Tb.Sp (Figure 5C) were unchanged by HU compared to NL controls. HU also led to deficits in some cortical parameters including increased Ma.Ar (Figure 6A) and Ec.Pm (Figure 6E) as well as decreased Ct.Ar (Figure 6B), and Ct.Th (Figure 6C) while Ps.Pm (Figure 6D) was unchanged in HU versus NL controls. Single housing, independent of HU, also led to modest deficits in some cancellous structural parameters including increased Tb.Sp (Figure 5C) and decreased Tb.N (Figure 5D). Further, the type of social environment did not significantly alter the response to HU in any of the cancellous (Figures 5A–D) and cortical (Figures 6A–E) μCT parameters measured, as there were no interaction effects evident by two-way ANOVA.

In C57BL/6J mice, there were no differences between socially housed groups versus NL controls in BV/TV (Supplementary Figure S5A), Tb.Sp (Supplementary Figure S5C), and Tb.N (Supplementary Figure S5D). In contrast, there was a ∼15% decrement in Tb.Th in the HU cohort relative to NL controls (Supplementary Figure S5B). Compared to NL controls, socially housed HU mice displayed a 16% increase in Ma.Ar (Supplementary Figure S6A). There was a modest increase of 9% in Ec.Pm (Supplementary Figure S6E) in socially housed HU mice versus NL controls. These were consistent with the trend seen for the same parameters in C57BL/6NJ mice. Ct. Ar was comparable between NL and HU groups of socially housed C57BL/6J mice (Supplementary Figure S6B) in contrast to the HU-induced deficits (although modest) observed in C57BL/6NJ mice (Figure 6B). Ps.Pm increased by 4% (Supplementary Figure S6D) although its biological relevance is unlikely due to the small magnitude of change. Overall, skeletal responses to HU of socially housed groups in both strains displayed similar trends and recapitulated expected disuse-induced changes in bone structure (Globus and Morey-Holton, 2016).

**FIGURE 7:**
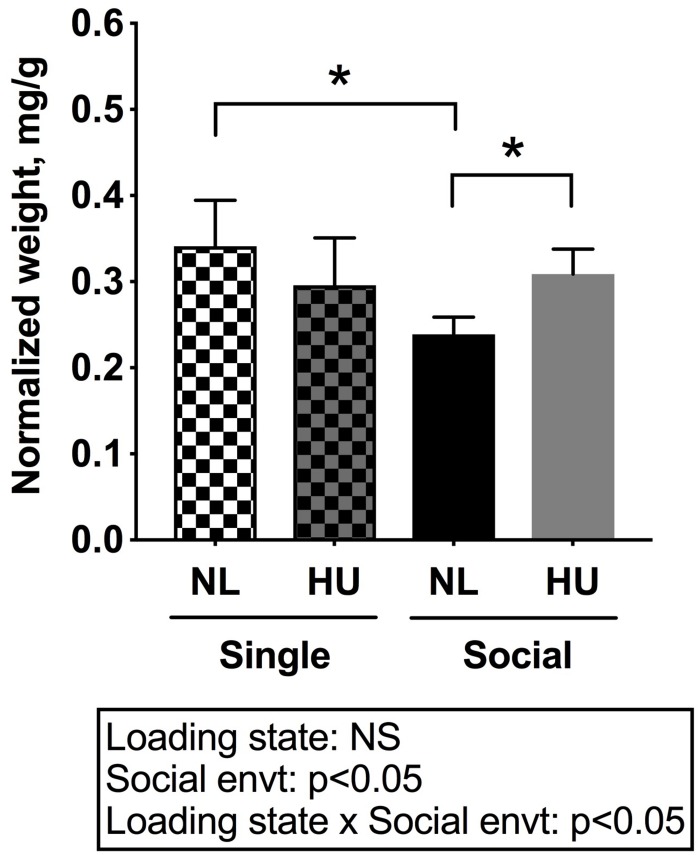
Left and right (combined) adrenal weights normalized to day 30 body weights of C57BL/6NJ mice. ^∗^Statistically significant at *p* < 0.05 using two-way ANOVA followed by Tukey *post hoc* test. NS: Not significant. Sample sizes: Single NL = 5; Single HU = 6; Social NL = 12; and Social HU = 12.

**FIGURE 8:**
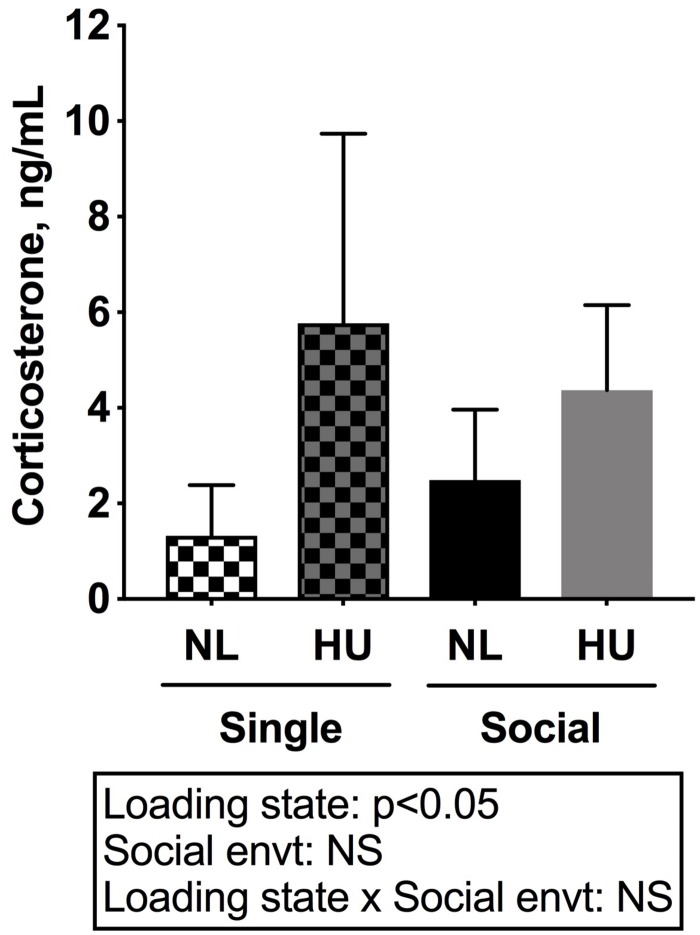
Plasma corticosterone levels of C57BL/6NJ mice. Two-way ANOVA with loglinear variance model was applied since unequal variance was observed. NS: Not significant. Sample sizes: Single NL = 8; Single HU = 5; Social NL = 12; and Social HU = 11.

To begin to gain insight into how social isolation during HU may impact the HPA axis, adrenal weights were measured at euthanasia and normalized to body weights (Figure 7) and plasma corticosterone levels were measured at the termination of the study (Figure 8). Adrenal hypertrophy can result from exposure to stressors such as social isolation (Gamallo et al., 1986; Westenbroek et al., 2005; Djordjevic et al., 2012) as well as crowding (Gamallo et al., 1986). In C57BL/6NJ mice, singly housed NL controls showed a 43% increase in normalized adrenal weight compared to socially housed NL controls (Figure 7). Singly and socially housed groups showed differential adrenal responses to HU. Whereas there were no statistically significant differences due to HU in singly housed mice, HU increased normalized adrenal weights by 29% compared to NL controls in socially housed mice. Singly housed HU and socially housed HU groups showed comparable values (Figure 7). In socially housed, C57BL/6J mice, HU did not cause a statistically significant increase in normalized adrenal weights (Supplementary Figure S7), although the trend was consistent with the differences observed in socially housed C57BL/6NJ mice (Figure 7). Plasma corticosterone levels at the termination of the experiment were evaluated in singly and socially housed C57BL/6NJ mice. HU led to increased levels in circulating corticosterone regardless of social environment (Figure 8).

**FIGURE 9:**
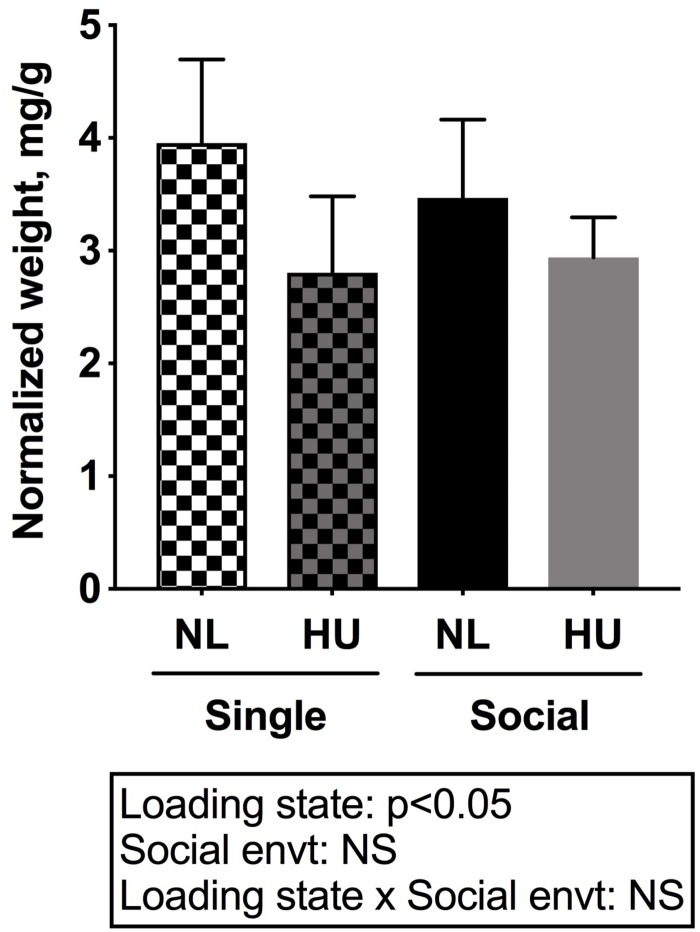
Spleen weights normalized to day 30 body weights of C57BL/6NJ mice. Two-way ANOVA was performed. NS: Not significant.

**FIGURE 10:**
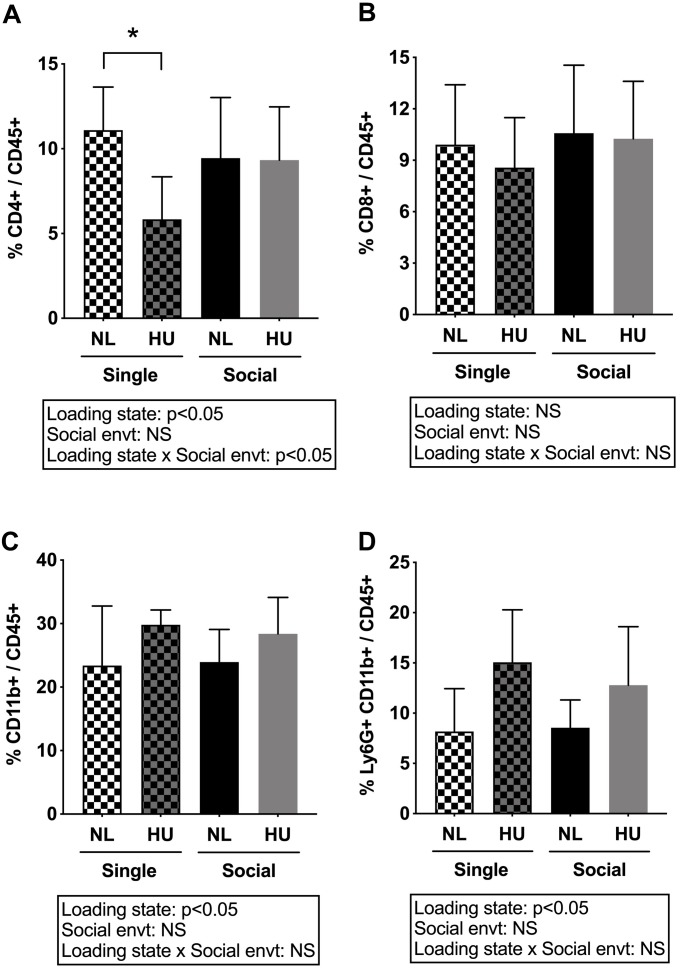
Percentages of white blood cell subpopulations in peripheral blood of C57BL/6NJ mice. **(A)** and **(B)** were analyzed by two-way ANOVA while **(C)** and **(D)** had unequal variances and were therefore analyzed by two-way ANOVA with loglinear variance model. NS: Not significant. ^∗^Statistically significant at *p* < 0.05 using Tukey *post hoc* test. NS: Not significant. Sample sizes: Single NL = 8; Single HU = 5; Social NL = 12; and Social HU = 12.

Spleen weights and blood cell populations also were examined to assess the contribution of social isolation on aspects of immunity during HU. In both C57BL/6J and C57BL/6NJ mice, HU led to a reduction in spleen weights (normalized to body weights) compared to NL controls (Figure 9 and Supplementary Figure S8). The type of social environment did not significantly alter splenic weights (Figure 9). To further evaluate immune effects, flow cytometric analyses of peripheral WBCs of C57BL/6NJ mice were performed to calculate the percentage of select leukocyte subpopulations relative to total WBC, using the common leukocyte antigen (CD45) as a general WBC marker (Figures 10A–D). Compared to NL controls, HU under single housing conditions led to a reduction in the percentage of CD4^+^ cells within CD45^+^ cells (Figure 10A) while this was not observed in HU under socially housed conditions. CD4^+^ immunoreactivity marks a subpopulation of T-helper cells. HU did not alter the percentage of CD8^+^/CD45^+^cells, a subpopulation of T-cytotoxic cells, in either social environment (Figure 10B). Further, the percentage of CD11b^+^ cells which broadly marks myeloid cells, including neutrophils and monocytes was increased by HU irrespective of social environment (Figure 10C). Similarly, HU led to an increased percentage of neutrophils marked by Ly6G^+^CD11b^+^ immunoreactivity (Figure 10D) regardless of social environment.

## Discussion

We have developed and validated a refinement to the NASA Ames HU model that accommodates paired housing of animals while retaining desirable features of the original design such as non-invasive attachment of the animal to the suspension harness and range of movement about the cage. An additional functionality to the current design includes individual access to food and water (not accessible to the other animal); thus, studies requiring customization of food or water as treatments can be readily conducted. Another feature of the current design includes the use of commercial-off-the-shelf (COTS) materials, which can help achieve significant cost and time savings over the previous standard version (Morey-Holton and Globus, 2002). These cage materials are compatible with typical cleaning and sterilization systems found in animal facilities. Finally, the cages occupy a smaller footprint since cage bottoms can be stacked for easy storage and improved transport. Overall, these changes to the design were intended to benefit investigators interested in conducting HU experiments.

Other groups have reported the use of paired housing HU systems for mice (Ferreira et al., 2011; Lloyd et al., 2012; Maurel et al., 2016). Similarities between the current design and a previously reported HU system (Ferreira et al., 2011) include the use of COTS components, individual access to food and water and physical contact of cephalad regions of pair housed animals. Notable differences include the manner by which tail attachment to the suspension apparatus is achieved. In the model developed by Ferreira et al. (2011), the tail is secured to the suspension apparatus via piercing of the tail with surgical wire which may prove useful should there be concerns about tail necrosis and/or animals coming down from suspension. In our hands, however, we have not encountered tail necrosis nor accidental release from suspension in our HU studies of short or long duration (up to 30 days) using the tail traction approach. Thus our system has the advantage of avoiding the stress associated with anesthesia and tissue injury from surgical procedures. In addition, the current HU system includes the use of a floor grate and bedding that covers the entirety of the cage floor in contrast to the Ferreira et al. (2011) design where no floor grate was employed and bedding partially covered the cage bottom. The choice of flooring (grate, bedding or bare) can potentially impact experimental results. Hence, variations in flooring across current HU systems is one consideration in experiment design and when comparing across studies. Another difference includes the range of movement about the cage. Our current design retains the pulley apparatus of the standard NASA Ames HU system (Morey-Holton and Globus, 2002), which generally allows for ambulation within most of the available cage area (close to half of the total cage area if socially housed). In contrast, the Ferreira et al. (2011) model secures the suspension apparatus via a fixed point which results in a range of movement restricted to a defined radius. Another group has conducted HU in mice housed in pairs although the design of the suspension apparatus prevented physical contact between animals (Lloyd et al., 2012; Krause et al., 2017), in contrast to our current design and the Ferreira et al. (2011) system which both allow cephalad regions of the animals to come in direct contact. Despite the prior existence of other HU designs that accommodate group housing, no other study has systematically assessed the contribution of the social environment in tissue responses to HU, a knowledge gap that we have begun to address here and highlights the novelty of this current study.

We validated this alternative social housing HU system by comparing it to the traditional single housing HU model. Standard housing controls were age- and sex-matched animals freely ambulating in standard mouse cages. Alternative controls have been used in other studies, such as restrained animals wherein the tail is attached to the suspension apparatus without elevating hindquarters (no musculoskeletal unloading, orthostatic suspension) (Gaignier et al., 2014; Rettig et al., 2019). One study reports that tail restraint in itself can lead to blood pressure and heart rate changes similar to that observed in singly housed HU rats (Tsvirkun et al., 2012), changes which were not observed in singly housed freely ambulating animals. Another variant to HU experiment design is to house ambulatory controls in HU cages (Huang et al., 2018). We opted for freely ambulating controls in standard vivarium cages. Although the literature is mixed, grate surfaces may alter HPA axis responses to stressors (Freed et al., 2008; Giral et al., 2011), and orthostatic suspension tail restraint, can itself cause stress responses (Tsvirkun et al., 2012). We recognize that selection of a different control group (e.g., restrained mice) may change some of the conclusions drawn from this study.

Males were not included in this study and it is well known that some behavioral and physiological responses to social isolation are sexually dimorphic (Caruso et al., 2017; Burke et al., 2018; Matsuda et al., 2018). For example, short-term isolation in female adults increases fear responses, as evaluated by contextual fear memory test, but not in age-matched males (Matsuda et al., 2018). Hence, it possible that some outcomes measured in this study may differ between males and females which can be addressed in future investigations.

In C57BL/6NJ mice, singly housed HU and NL controls had comparable body weights throughout the experiment while socially housed HU animals displayed a modest decrease in body weights compared to their corresponding controls. The reason for the persistent difference in body weights in the socially housed groups requires further study (food intake could not be reliably measured). It is possible that behaviors unique to the NL and HU social housing groups may play a role in these differences. Novel behaviors such as nestlet manipulation could have led to an overall increase in activity levels in socially housed HU animals compared to NL controls, and therefore may result in lower body weights of the socially housed HU mice. Unlike C57BL/6NJ mice, socially housed HU and NL control C57BL/6J animals had comparable body weights. These findings from C57BL/6J females are consistent with the report of Ferreira et al. (2011) using the same strain, sex and age at onset of HU (personal communication with Dr. M. Brown). The underlying basis for these observations is not yet understood, although the different metabolic phenotypes of these two strains (Fisher-Wellman et al., 2016) may have contributed to the discrete effects of social housing HU on body weights.

In both strains tested (C57BL/6J and C57BL/6NJ), housing HU mice in social pairs led to the expected decrements in bone microstructural parameters and soleus muscle weights. The magnitudes of musculoskeletal deficits induced by HU between singly and socially housed animals were similar. One interpretation of these findings is that loss of mechanical loading and/or fluid shifts contribute primarily to the osteopenia and sarcopenia caused by the traditional model of simulated weightlessness, while social isolation does not appear to significantly impact these outcomes. However, since HU regardless of social environment led to increases in circulating corticosterone levels, we could not completely rule out the possible contribution of neuroendocrine stress responses (from other sources) to these observed musculoskeletal deficits. Further, direct measures of muscle function (e.g., hindlimb grip strength) and bone mechanical properties (e.g., three point bending of tibia or femur) were not conducted due to logistical constraints. Hence, it remains to be elucidated whether the social environment can differentially impact muscle function and bone strength or whether magnitudes of the deficits (if any) will be similar to bone and muscle outputs in this study.

Immune system dysregulation occurs during both short and long duration spaceflight missions (∼6 months) (Crucian et al., 2015, 2018). Spaceflight-induced alterations in immune function include reactivation of herpes viruses (Mehta et al., 2017) as well as changes in the distribution of immune cell populations (Gridley et al., 2009; Morukov et al., 2011). In this study, simulated weightlessness by HU caused a decrement in spleen weight regardless of social environment. Our findings on HU-induced spleen atrophy is consistent with that observed in autoimmune disease (Dillon et al., 1982) and often in spaceflight (Gridley et al., 2003, 2013), while decrements in spleen mass have long been associated with chronically elevated levels of corticosterone and stress (Batuman et al., 1990; Voorhees et al., 2013). Splenectomy compromises the immune system by increasing susceptibility to infections (Sullivan et al., 1978). Compromised immunity in asplenic individuals are primarily seen in reduced Ig class switching and B cell immunity, and although not studied in this report, reduced pre- and pro-B cell populations, along with reduced spleen weight, also have been described in bone loss models (Panaroni et al., 2015). However, it must be noted that other factors apart from immune perturbations could give rise to changes in spleen weight. While the spleen is regarded as a secondary lymphoid organ, it serves additional functions in terms of RBC maintenance and iron storage (Pivkin et al., 2016). Furthermore, the spleen also can accommodate fluid pressure shifts by widening or narrowing internal splenic blood vessels in response to signaling atrial natriuretic factor (ANF) from cardiac myocytes (Kaufman, 1992; Sultanian et al., 2001; Kaufman and Levasseur, 2003), which may serve an important role during microgravity-related fluid pressure shifts.

In contrast to our findings in the musculoskeletal system, we observed that some aspects of the immune response to HU can be differentially impacted by the social environment. In C57BL/6NJ mice, HU reduced the percentage of CD4^+^/CD45^+^ cells (subpopulation of T-helper cells) compared to NL controls in singly housed mice, but not in socially housed mice. These findings were consistent with other reports of decreased T cell numbers in studies using singly housed mice as well as increased susceptibility to infections in crew members during and after flight (Gridley et al., 2009; Crucian et al., 2015, 2018; Mehta et al., 2017). Differential changes in immune cell populations in HU mice have been reported by others but this was not always observed, with different timepoints of collection likely playing a factor in these outcomes. For example, HU (4 days) increased blood neutrophil counts (Sanzari et al., 2013) while HU (21 days) reduced B cells and decreased T-helper (CD4^+^)/T-cytotoxic (CD8^+^) ratio of mouse splenocytes (Gaignier et al., 2014). Others also have reported reduced CD4^+^ cells in HU (singly housed animals) (Wei et al., 2003; Wang et al., 2007), suggesting disrupted immunity. One important implication of our findings for future spaceflight missions is that facilitating social interactions can potentially mitigate some of the spaceflight-induced immune impairments. In addition, studies report elevation of absolute WBCs and neutrophils in astronauts upon landing (Kaur et al., 2004), along with reduced T cell function during short-term (Stowe et al., 2011) and long-term (Crucian et al., 2015) spaceflight, lasting weeks and months, respectively. HU regardless of social environment increased the percentage of myeloid populations (CD11b^+^) and a subset within this population, specifically neutrophils (Ly6G^+^CD11b^+^). Although there are other possible interpretations, these findings suggest that some immune cell populations (e.g., neutrophils) may be more sensitive to perturbations in gravitational loading and/or fluid shifts than others. Due to logistical limitations, characterization of immune cell populations on C57BL/6J mice was not conducted. Further studies are needed to evaluate strain-dependence of these immune responses. Taken together, our findings indicate that HU and social isolation can both have discrete and interactive effects on the immune system.

Our findings also indicate that the social environment can differentially impact some aspects of the HPA axis in response to HU. In C57BL/6NJ mice, singly housed NL controls showed increased adrenal weights compared to socially housed controls consistent with findings of others (Djordjevic et al., 2012). In the present study, there were no differences between singly housed NL control and HU groups, suggesting that HU did not exacerbate any adrenal weight changes incurred by social isolation and vice versa. Adrenal weights were increased in socially housed HU cohorts compared to corresponding NL controls. Taken together, our results are consistent with the possibility that both prolonged simulated weightlessness and social isolation can induce chronic stress (adrenal hypertrophy) although the effects of each factor are not additive. These findings also suggest that social isolation can mask the effects of HU on adrenal hypertrophy.

The socially housed animals (NL and HU) in this study had no prior history as cagemates with the exception of one HU pair. To minimize the contribution of stress responses from unfamiliar cagemates and/or a new cage environment, animals were acclimated with their new cagemates for 3 days prior to HU. However, it is conceivable that stress from housing with an unfamiliar individual may have contributed to some of the HPA axis results, although assessment of the values for the pair with a longer history of co-habitation did not show any obvious differences compared to the other 10 socially housed mice.

Other studies suggest benefits from less frequent cage and bedding changes which is thought to facilitate the maintenance of environmental olfactory cues thereby minimizing stress. For example pup mortality in C57BL/6J mice was reduced when cage changes were performed every 14 or 21 days instead of every 7 days (Reeb-Whitaker et al., 2001). In our experience, daily changes of nestlets were necessary to minimize soiling and to prevent the buildup of ramp-like structures that may lead to undesirable loading of the hindlimbs in HU animals. Cages and bedding were changed weekly which allows for retention of olfactory cues.

There were no significant correlations between body weights and normalized adrenal gland weights nor plasma corticosterone levels (data not shown). Hence, we reason that it is less likely that the observed body weight differences in singly and socially housed C57BL/6NJ HU animals can be attributed solely to HPA axis reactivity. There were no differences in adrenal gland weights in socially housed HU and control C57BL/6J mice in our study, consistent with findings of Ferreira et al. (2011) from pair-housed HU (for 28 days) in females of the same strain and age. Collectively these results indicate that HPA axis responses to HU can be strain-dependent. In this current study, HU regardless of social environment increased circulating corticosterone levels in C57BL/6NJ mice, although there was wide within-group variance. Taken together, our findings suggest that both social isolation and HU in females can lead to responses typically associated with HPA axis activation such as increased adrenal weights and corticosterone levels.

The HPA axis interacts with the hypothalamic-pituitary-gonadal (HPG) axis (Mastorakos et al., 2006). Investigating the effects of HPA on the HPG axis was outside the scope and hypotheses of this study. However, we recognize that alterations in neuroendocrine signals can have an impact on ovarian function which in turn can drive skeletal changes independent of unloading. Estrogen deficiency (Roggia et al., 2001) and unloading (Shahnazari et al., 2012) each can activate bone remodeling. When combined (e.g., HU or spaceflight ovariectomized animals) (Turner et al., 1998; Keune et al., 2015), unloading caused bone loss greater than that from ovariectomy alone, indicating bone loss is multi-factorial and includes systemic and local inputs. The effects of long duration skeletal unloading on ovarian function is not well understood. In rats, HU tends to reduce estrogen levels and cause prolonged diestrus, changes associated with elevated levels of corticosterone (Tou et al., 2004) together with the expected osteopenia (Tou et al., 2005). Further studies are needed to elucidate the interactions of HPA and HPG axes with musculoskeletal unloading and how it impacts responses to microgravity.

Tight regulation of the HPA axis is critical for the maintenance of tissue health, including the immune system (Bellavance and Rivest, 2014). Elevated levels of glucocorticoids resulting from chronic stress, exogenous therapy, or endocrine disorders can lead to immune dysfunction and increased osteoclast activity that contributes to bone loss (Frenkel et al., 2015). Individuals with Cushing’s disease, a neuroendocrine disorder characterized by over-production of adrenocorticotropic hormone (ACTH) and therefore cortisol, display immune impairments, such as decreased CD4^+^ populations, which we also observed in singly housed HU mice. Neutrophils highly express 11β-hydroxysteroid dehydrogenase type 1 (11β-HSD1), which converts cortisone and 11-dehydrocorticosterone into their respective active forms, cortisol and corticosterone. Neutrophils have been reported to be the main population of immune cells expressing 11β-HSD1 in an inflammation model of peritonitis (Coutinho et al., 2016). Our findings indicate that HU regardless of social environment led to increased percentage of neutrophils and circulating corticosterone levels. We postulate that the increase in neutrophils could have contributed to the elevation in circulating corticosterone levels observed during HU. It is currently unclear whether the HPA axis contributes to the immune changes observed in HU or vice versa. Establishing the direct link between the HPA axis and the observed HU-induced immune changes in singly and socially housed animals needs further study.

Percent CD4^+^ population and adrenal weights are the two outcomes that show differences between singly and socially housed animals. Although comprising a small subset of the outcomes measured in this study, changes in CD4^+^ population (T-helper) and HPA axis signals can have a profound impact on immunity and other biological processes. Taken together, our findings have important implications for the use of the HU model to simulate exposure to the spaceflight environment. The possible influence of social isolation and HPA axis responses should be taken into account when using and interpreting results obtained from the traditional HU model. In addition, the suitability of these two models in meeting specific experimental objectives needs to be considered. For instance, studies focusing on multiple aspects of the spaceflight environment such as social isolation stress and microgravity, may benefit from employing the traditional single housing HU model. In contrast, investigations that aim to define physiological changes that are gravity-dependent (excluding social isolation stress) may be better conducted using the social housing HU model.

In summary, we developed and validated an alternative social housing HU system that retains desirable features of the traditional NASA Ames HU system. We found that deficits in muscle and bone microarchitecture observed in HU mice were fundamentally a response to changes in gravitational loading (i.e., hindquarter disuse and fluid shifts) while components of the immune system and HPA axis were differentially impacted by the social environment during HU. While not in absolute solitude, social confinement is experienced in launch/landing and on-board the ISS and is therefore of high relevance to spaceflight, especially for long duration missions. The availability of both single and social housing HU models can facilitate studies that aim to distinguish the effects of social interactions on the HPA axis and immune system, thereby expanding the utility of the HU model for studying how the spaceflight environment impacts organ systems.

## Data Availability

All datasets generated for this study are included in the manuscript and/or the Supplementary Files.

## Ethics Statement

Animals experiments were carried out in accordance with the recommendations of Guide for the Care and Use of Laboratory Animals, 8th edition (National Research Council (U.S.) Committee for the Update of the Guide for the Care and Use of Laboratory Animals, Institute for Laboratory Animal Research (U.S.) and National Academies Press (U.S.), 2011). All animal experiments were conducted with prior approval from the NASA Ames Institutional Animal Care and Use Committee (IACUC).

## Author Contributions

CT, AS, and RG conceived and designed the study. CT, AS, ST, SS, AP, LR, and ML collected the data. RG, JA, CT, and ST conceptualized and designed the social housing HU cages. CT, ST, SS, ML, AS, LR, and AP analyzed the data. CT, RG, AS, AP, AR, LR, and SB interpreted the data. CT wrote the first draft of the manuscript. RG and AP wrote sections of the manuscript. All authors contributed to the manuscript revision, read, and approved the submitted version.

## Conflict of Interest Statement

The authors declare that the research was conducted in the absence of any commercial or financial relationships that could be construed as a potential conflict of interest.
