# Supplementary material for: Influence of Social Isolation During Prolonged Simulated Weightlessness by Hindlimb Unloading
Source: Front Physiol. 2019 Sep 13;10:1147. doi: 10.3389/fphys.2019.01147 (PMC6753329; doi:10.3389/fphys.2019.01147)
Supplement: Supplementary file 1 [file Data_Sheet_1.pdf]

## Supplementary Methods

**C57BL/6J study design and sample collection.** Sixteen-week old C57BL/6J female mice underwent hindlimb unloading (HU) as per procedures described for C57BL6/NJ in the Methods section. C57BL/6J mice were assigned to one of two groups: HU under socially-housed conditions or normally ambulating (NL) controls housed in standard vivarium cages. There were no single housing experimental groups due to logistical constraints. The same tissue collection and processing scheme was performed as described in the Methods section. Unless otherwise stated, sample sizes were N=8 for HU and N=7 for the NL group. The social HU group began with N=8 but one animal was removed from the study due to a decline in body weight.

**Statistical analysis of C57BL/6J data sets.** A repeated measures ANOVA was performed on C57BL/6J mice body weight data. Student's t-test was used for statistical analyses of all other datasets from this mouse strain. A heteroskedastic t-test was applied if the datasets showed unequal variance. JMP software version 13.1.0 (SAS Institute Inc) was used to conduct statistical analyses. Data shown are mean +/- S.D.

**Video recording and scoring of C57BL/6NJ mice behavior.** On day 28 of HU, video footage was recorded (30 fps) for a period of 24 hours (12:12 light:dark cycle) using a Lorex model LX1080-166W 16-channel HD camera and DVR system. Three 15-minute periods during the dark cycle were analyzed to quantify manipulation of the nestlet enrichment, termed "novel nestlet behavior", which was defined as the action of passing a nestlet to a cagemate or removing it. Trained coders reviewed the video segments in real time and in slow motion (frame-by-frame). To determine inter-rater concordance, the selected behaviors were independently scored by three evaluators, achieving an average inter-rater reliability of $R>0.97$. Due to obvious procedure differences between loading conditions, coders were unable to score the behavior of HU and NL mice blinded to treatment.
