# Supplementary figures and images for: Influence of Social Isolation During Prolonged Simulated Weightlessness by Hindlimb Unloading
Source: Front Physiol. 2019 Sep 13;10:1147. doi: 10.3389/fphys.2019.01147 (PMC6753329; doi:10.3389/fphys.2019.01147)

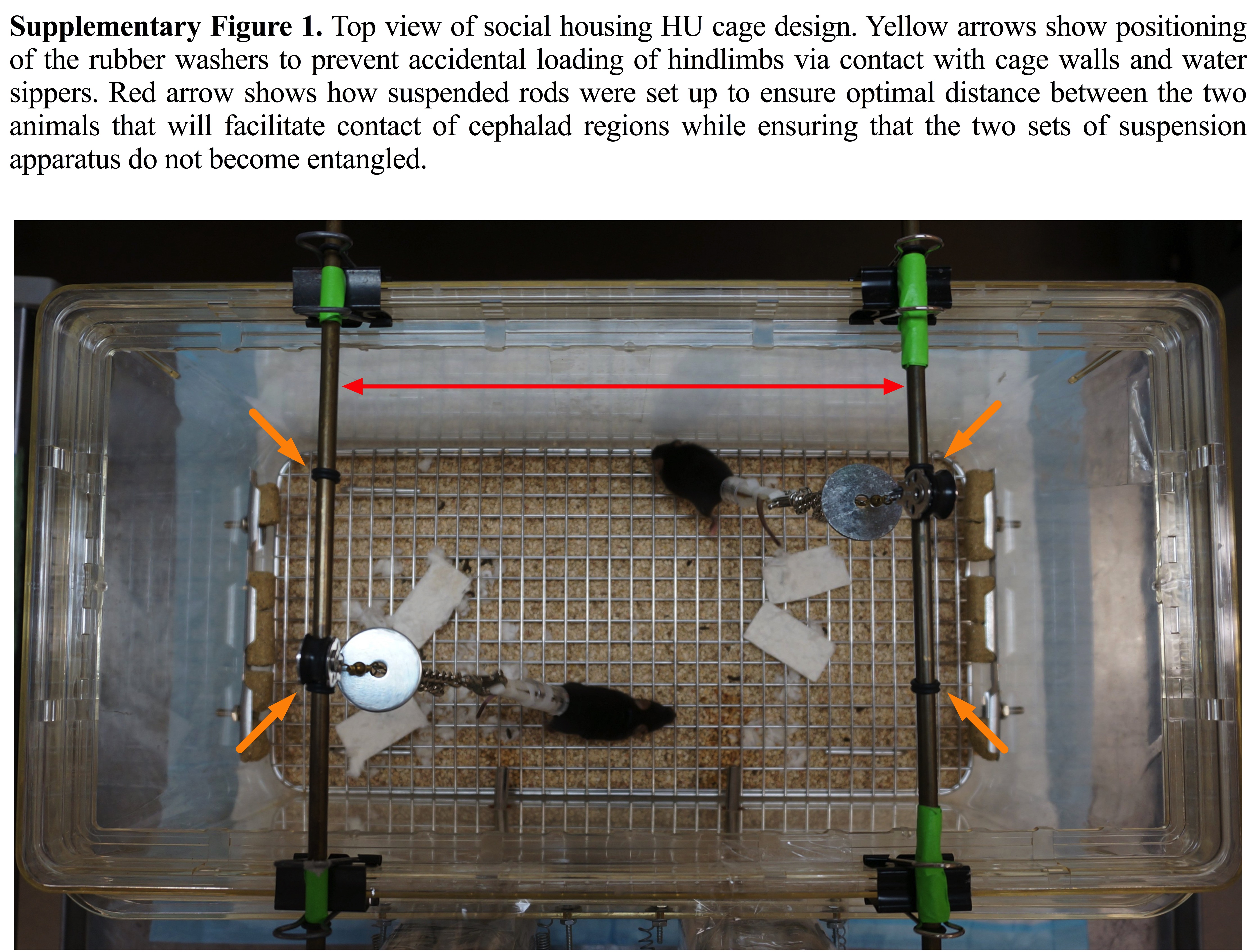

Supplement: Supplementary file 2 [file Image_1.JPEG]

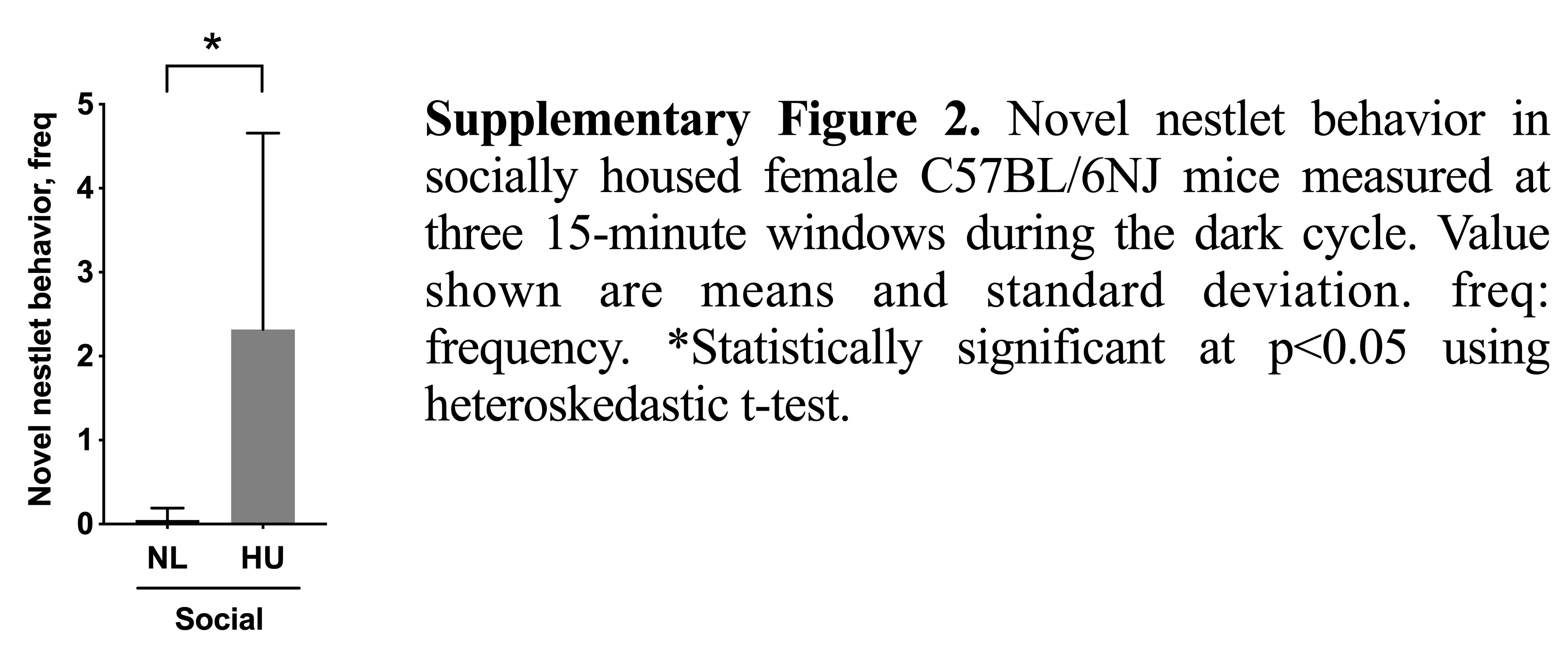

Supplement: Supplementary file 3 [file Image_2.jpg]

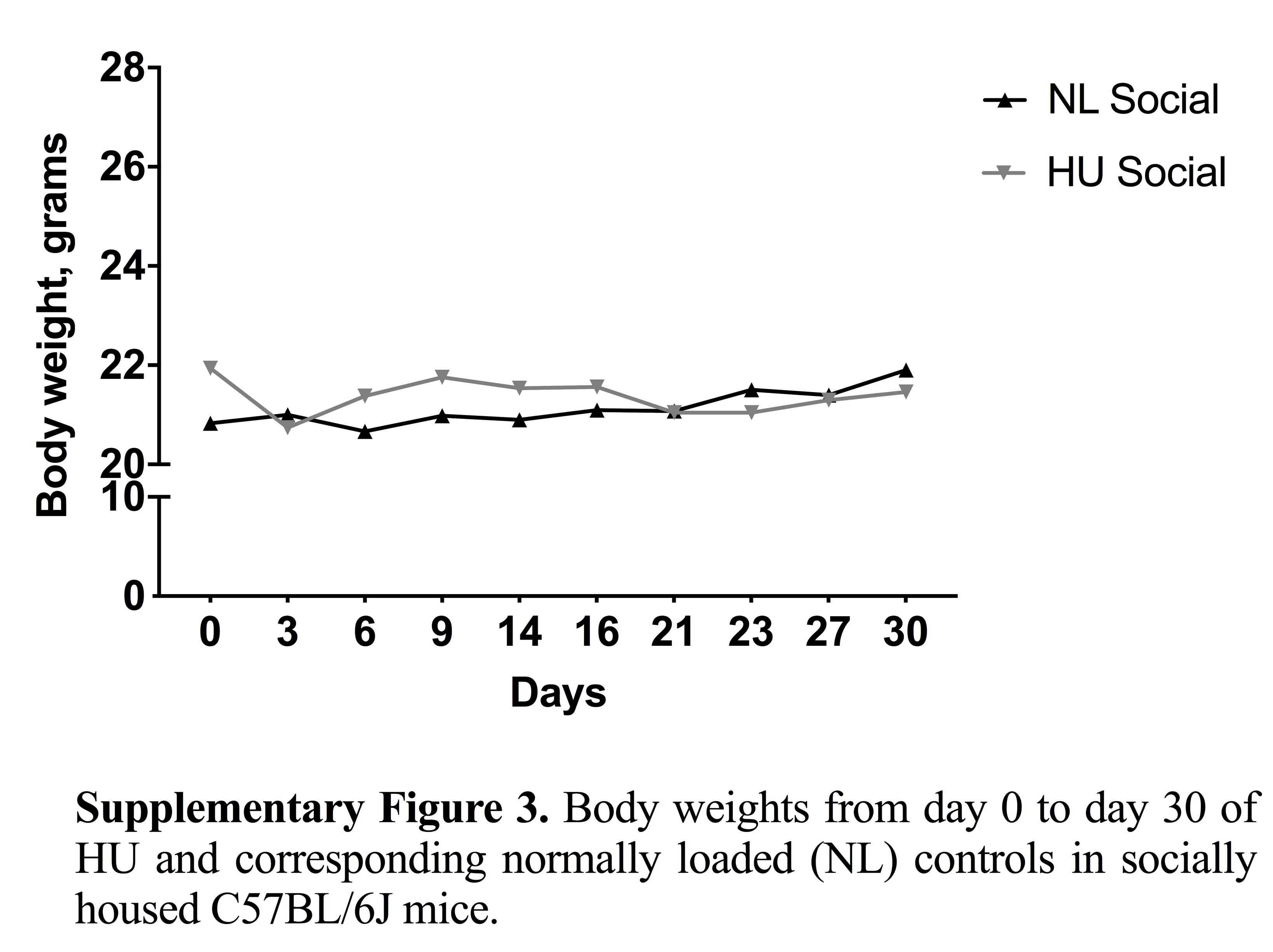

Supplement: Supplementary file 4 [file Image_3.JPEG]

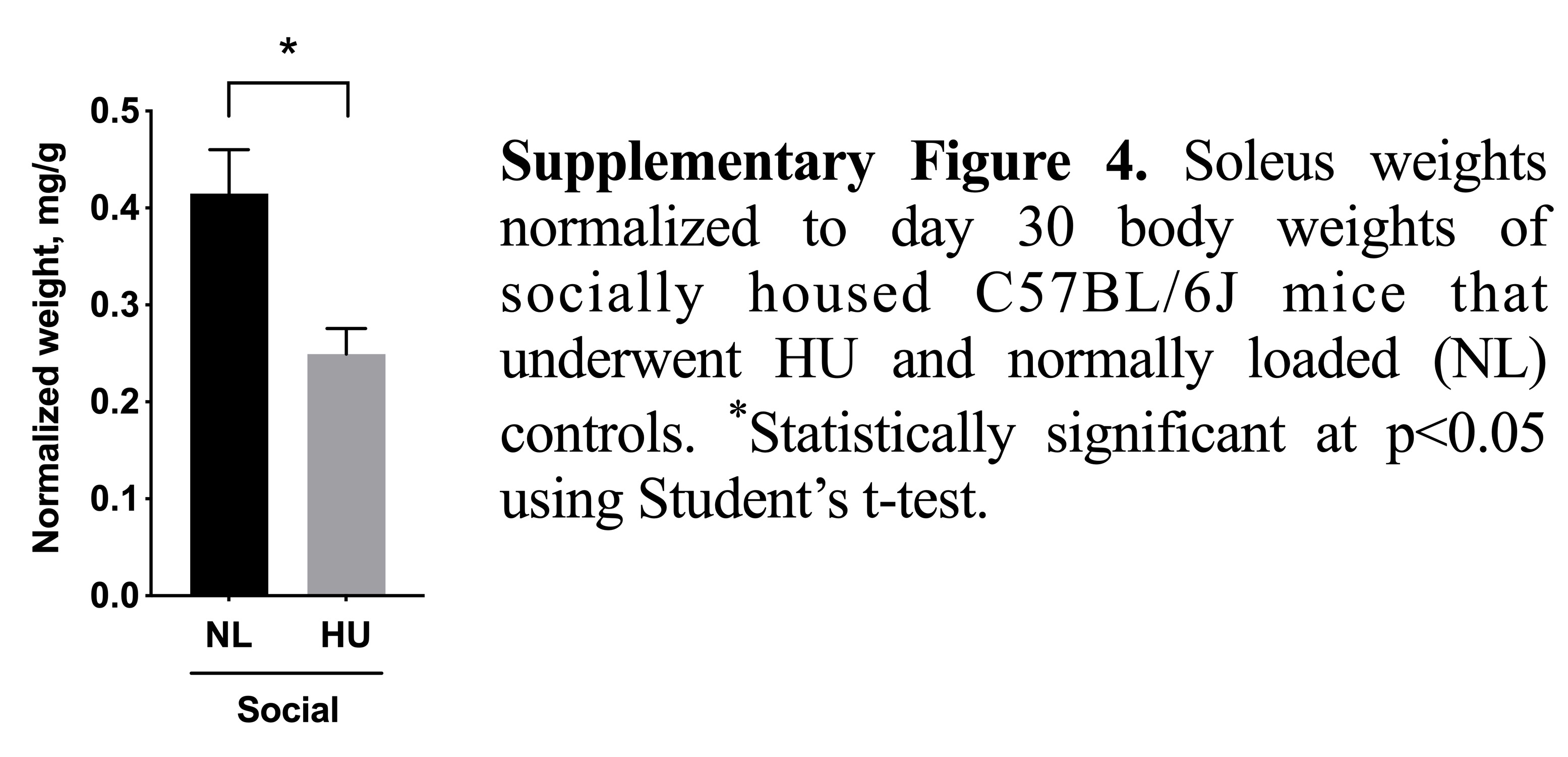

Supplement: Supplementary file 5 [file Image_4.jpg]

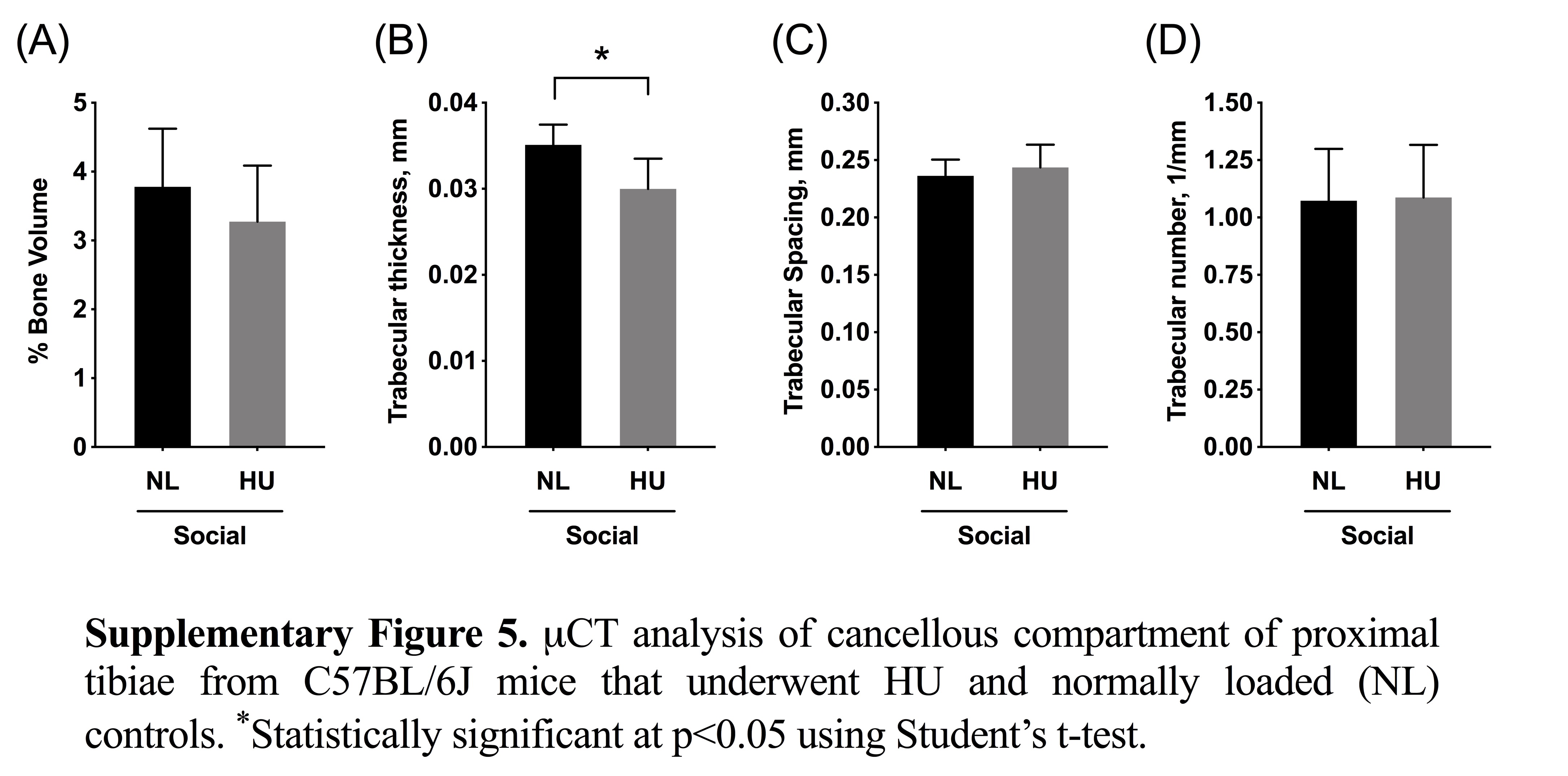

Supplement: Supplementary file 6 [file Image_5.JPEG]

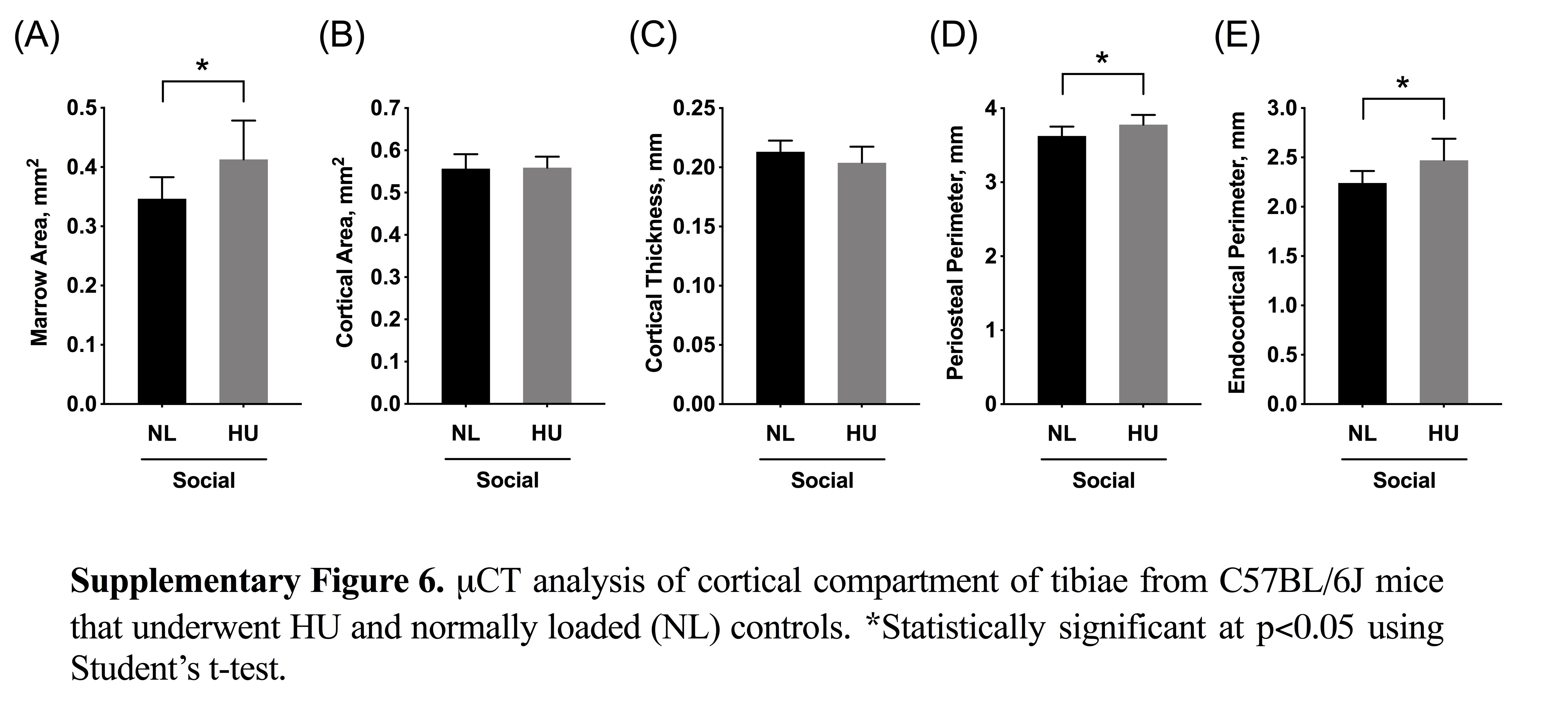

Supplement: Supplementary file 7 [file Image_6.JPEG]

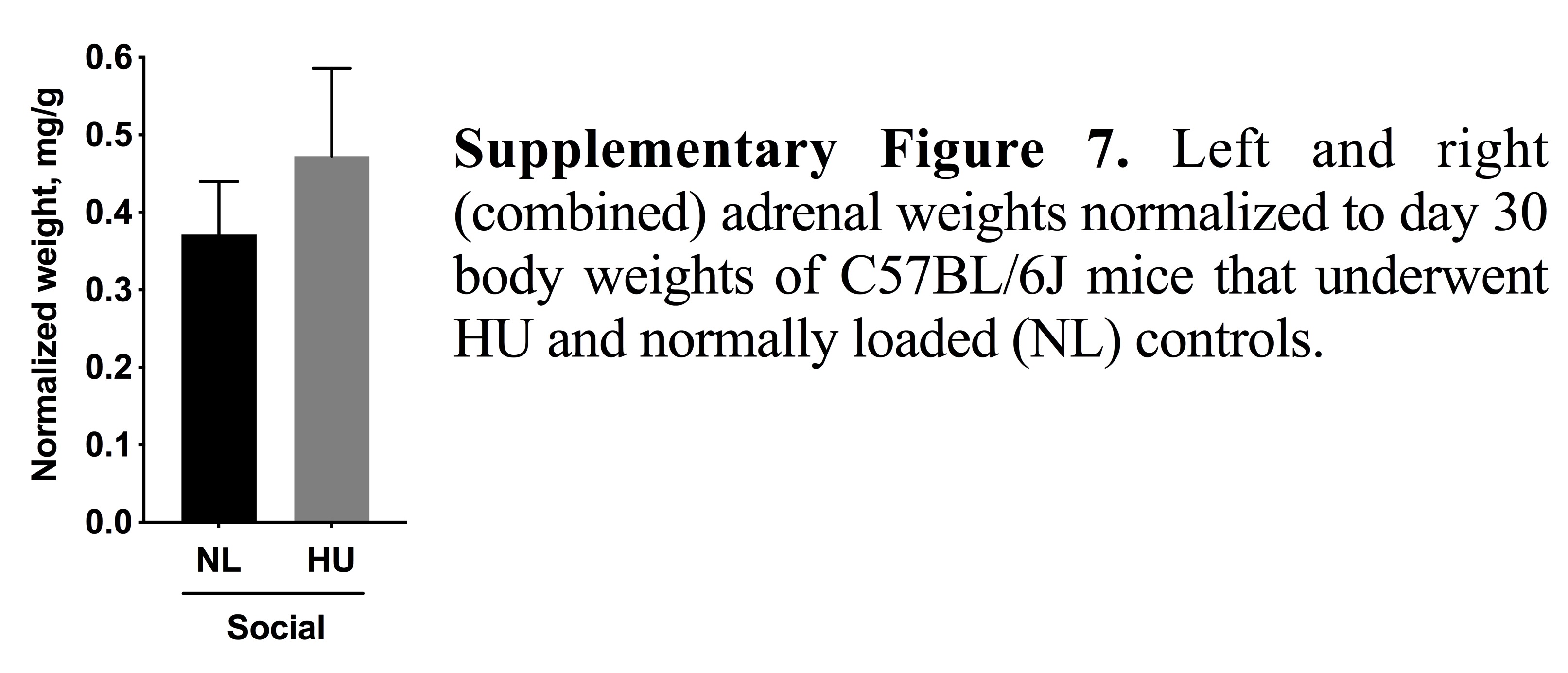

Supplement: Supplementary file 8 [file Image_7.JPEG]

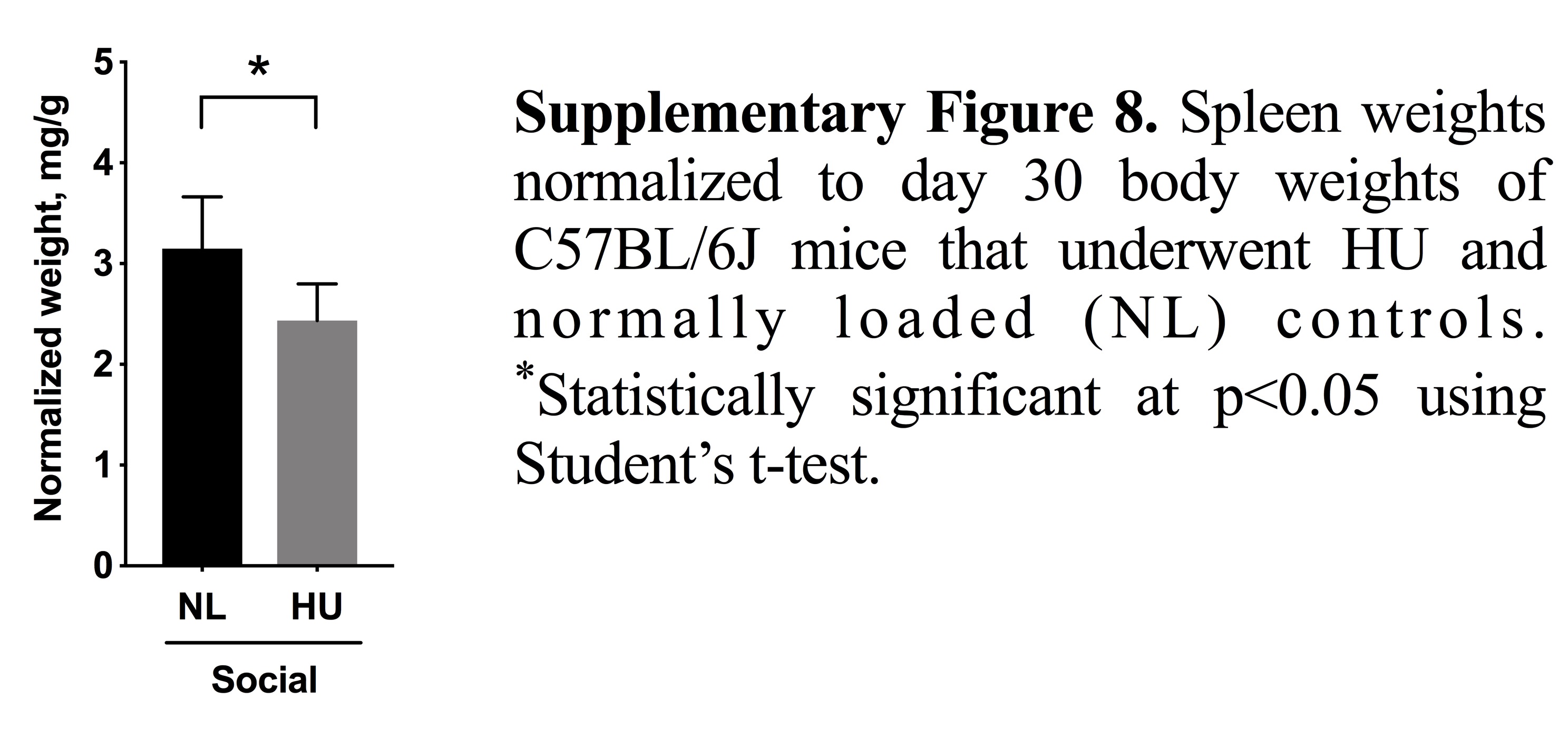

Supplement: Supplementary file 9 [file Image_8.JPEG]
