# Supplementary material for: Influence of Social Isolation During Prolonged Simulated Weightlessness by Hindlimb Unloading
Source: Front Physiol. 2019 Sep 13;10:1147. doi: 10.3389/fphys.2019.01147 (PMC6753329; doi:10.3389/fphys.2019.01147)
Supplement: Supplementary file 10 [file Table_1.DOCX]

**Supplementary Table 1.** Mean body weights (grams) of female C57BL/6NJ mice taken at multiple time points during HU and corresponding NL controls. SD: Standard deviation. Sample sizes for each group are indicated in the top column. Note that an additional four animals used in the NL single group (total N=8) only had body weight measures for the day 30 timepoint and are therefore not shown in the table below.

|  | **NL Single, N=4** | | **HU Single, N=6** | | **NL Social, N=12** | | **HU Social, N=12** | |
| --- | --- | --- | --- | --- | --- | --- | --- | --- |
| **Days** | **Mean** | **SD** | **Mean** | **SD** | **Mean** | **SD** | **Mean** | **SD** |
| 0 | 22.77 | 0.73 | 23.83 | 1.31 | 23.73 | 1.43 | 22.94 | 1.22 |
| 7 | 22.84 | 1.20 | 23.27 | 1.29 | 23.31 | 0.95 | 21.95 | 1.25 |
| 14 | 23.04 | 1.03 | 23.39 | 1.81 | 23.71 | 0.76 | 21.96 | 1.14 |
| 21 | 23.07 | 1.64 | 23.12 | 1.78 | 23.98 | 1.29 | 22.38 | 1.43 |
| 28 | 23.85 | 1.29 | 23.73 | 1.40 | 24.43 | 1.19 | 22.58 | 1.11 |
| 30 | 23.89 | 1.30 | 24.26 | 1.02 | 24.44 | 1.10 | 23.09 | 1.26 |
