# Supplementary material for: Influence of Social Isolation During Prolonged Simulated Weightlessness by Hindlimb Unloading
Source: Front Physiol. 2019 Sep 13;10:1147. doi: 10.3389/fphys.2019.01147 (PMC6753329; doi:10.3389/fphys.2019.01147)
Supplement: Supplementary file 11 [file Table_2.DOCX]

**Supplementary Table 2.** Mean body weights (grams) of female C57BL/6J mice taken at multiple time points during HU and corresponding NL controls. SD: Standard deviation. Sample sizes for each group are indicated in the top column.

|  | **NL Social, N=8** | | **HU Social, N=7** | |
| --- | --- | --- | --- | --- |
| **Days** | **Mean** | **SD** | **Mean** | **SD** |
| 0 | 20.83 | 1.64 | 21.94 | 1.73 |
| 3 | 21.00 | 1.44 | 20.74 | 2.22 |
| 6 | 20.67 | 1.58 | 21.38 | 1.39 |
| 9 | 20.98 | 1.57 | 21.76 | 1.47 |
| 14 | 20.90 | 1.33 | 21.54 | 1.62 |
| 16 | 21.10 | 1.36 | 21.56 | 1.61 |
| 21 | 21.08 | 1.44 | 21.04 | 1.97 |
| 23 | 21.51 | 1.70 | 21.05 | 2.04 |
| 27 | 21.40 | 1.28 | 21.30 | 2.13 |
| 30 | 21.90 | 1.23 | 21.46 | 1.84 |
