# Supplementary material for: Influence of Social Isolation During Prolonged Simulated Weightlessness by Hindlimb Unloading
Source: Front Physiol. 2019 Sep 13;10:1147. doi: 10.3389/fphys.2019.01147 (PMC6753329; doi:10.3389/fphys.2019.01147)
Supplement: Supplementary file 12 [file Table_3.DOCX]

**Supplementary Table 3.** Wet tissue weights (in milligrams) of C57BL/6NJ mice at day 30 of HU and corresponding NL controls. Left and right adrenals were pooled for weighing.

|  | **Soleus** | | **Adrenals** | | **Spleen** | |
| --- | --- | --- | --- | --- | --- | --- |
| **Group** | **Mean** | **SD** | **Mean** | **SD** | **Mean** | **SD** |
| NL Single | 9.35 | 1.77 | 8.56 | 1.6 | 9.60 | 1.14 |
| HU Single | 5.10 | 0.087 | 7.18 | 1.38 | 6.77 | 1.51 |
| NL Social | 8.27 | 0.078 | 5.83 | 0.55 | 8.48 | 1.78 |
| HU Social | 5.04 | 0.068 | 7.07 | 0.69 | 6.80 | 0.98 |
