# Supplementary material for: Influence of Social Isolation During Prolonged Simulated Weightlessness by Hindlimb Unloading
Source: Front Physiol. 2019 Sep 13;10:1147. doi: 10.3389/fphys.2019.01147 (PMC6753329; doi:10.3389/fphys.2019.01147)
Supplement: Supplementary file 13 [file Table_4.DOCX]

**Supplementary Table 4.** Wet tissue weights (in milligrams) of C57BL/6J mice at day 30 of HU and NL controls. Left and right adrenals were pooled for weighing.

|  | **Soleus** | | **Adrenals** | | **Spleen** | |
| --- | --- | --- | --- | --- | --- | --- |
| **Group** | **Mean** | **SD** | **Mean** | **SD** | **Mean** | **SD** |
| NL Social | 9.09 | 1.13 | 8.14 | 1.57 | 66.2 | 3.9 |
| HU Social | 5.37 | 0.90 | 10.09 | 2.27 | 52.6 | 1.3 |
